# Panniculitis-like T-cell-lymphoma in the mesentery associated with hemophagocytic syndrome: autopsy case report

**DOI:** 10.1186/s13000-019-0854-9

**Published:** 2019-07-17

**Authors:** Jan Hrudka, Václav Eis, Josef Heřman, Zuzana Prouzová, Andreas Rosenwald, František Duška

**Affiliations:** 1 Department of Pathology, Charles University, 3rd Faculty of Medicine, Charles University and Kralovske Vinohrady University Hospital, Prague, Czech Republic; 2 Department of Pathology and Molecular Medicine, 2nd Faculty of Medicine, Charles University and Motol University Hospital, Prague, Czech Republic; 3 Department of Pathology, Julius-Maximilian University, Faculty of Medicine, Würzburg, Germany; 4 Department of Anaesthesiology and Intensive Care, Charles University, 3rd Faculty of Medicine, Charles University and Kralovske Vinohrady University Hospital, Prague, Czech Republic

**Keywords:** Panniculitis, T-cell lymphoma, Mesentery, Hemophagocytosis, Lymphohistiocytosis

## Abstract

**Background:**

Panniculitis-like T-cell lymphoma is an uncommon type of non-Hodgkin lymphoma, occurring usually in the form of nodules within the subcutaneous fat tissue of the extremities or trunk. In the literature, subcutaneous panniculitis-like T-cell lymphoma (SPTCL) is described as a distinct type of T-cell lymphoma with a variable clinical behavior, depending on molecular phenotype of T-cell receptor (TCR) and on the presence or absence of hemophagocytic syndrome.

**Case presentation:**

We present a bioptic and autoptic case of a 65-years old Caucasian man with panniculitic T-cell lymphoma with morphological and immunohistochemical features of SPTCL, limited to the retroperitoneal and mesenteric mass, i.e. without any cutaneous involvement, and associated with severe hemophagocytic lymphohistiocytosis.

**Conclusion:**

A panniculitic T-cell lymphoma with morphological and molecular features of SPTCL, which is limited to mesentery, i.e. does not involve subcutaneous fat, seems to be exceedingly rare.

## Background

Subcutaneous panniculitis-like T-cell lymphoma (SPTCL) is a cytotoxic T-cell lymphoma that preferentially infiltrates subcutaneous adipose tissue [1]. It is a rare form of lymphoma, accounting for < 1% of all non-Hodgkin lymphomas. SPTCL can occur at any age, with 20% of patients being < 20 years of age [2]. The diagnosis of SPTCL is challenging. Half of the patients with SPTCL present with non-specific clinical symptoms incl. weight loss, low-grade fever and general malaise, whilst the rest may only have local signs [1, 3]. Locally, there are multiple subcutaneous nodules or plaques ranging from 5 mm to several centimeters in size. They most commonly occur in the subcutaneous tissue of the extremities or trunk. Other locations are rare [4], but may include the mesentery [5, 6]. Lymph node or bone marrow involvement is usually absent [2]. The differential diagnosis includes panniculitis, either unspecific [7] or associated with lupus [3]. Laboratory abnormalities frequently include cytopenia and elevated liver function tests. Panniculitic T-cell lymphoma may be associated with hemophagocytic syndrome in 17–45% cases, depending on molecular phenotype of T-cell receptor (TCR) of tumor cells [3].

Even a biopsy may not lead to a straightforward diagnosis. There are lymphocytic infiltrates involving the fat lobules, but usually sparing the septa. The lymphoma cells vary in size, have irregular and hyperchromatic nuclei. The rimming of the neoplastic cells surrounding individual fat cells is a helpful diagnostic clue. Admixture of reactive histiocytes is usually found in the areas of fat infiltration and destruction [1]. In immunohistochemistry, the neoplastic cells in SPTCL express α/β cytotoxic T-cell phenotype, including CD8 (cluster of differentiation), TIA1 (T-cell intracellular antigen 1), granzyme B and perforin, but not CD56 and CD4 [1].

In this paper, we present case of a patient with panniculitis-like cytotoxic T-lymphoma of the mesentery, with microscopic and immunohistochemical features of SPTCL and both clinical and histopathological signs of severe hemophagocytic syndrome, but without any (sub)cutaneous involvement, which we believe is exceedingly rare.

## Case presentation

### Clinical history

**Fig. 1:**
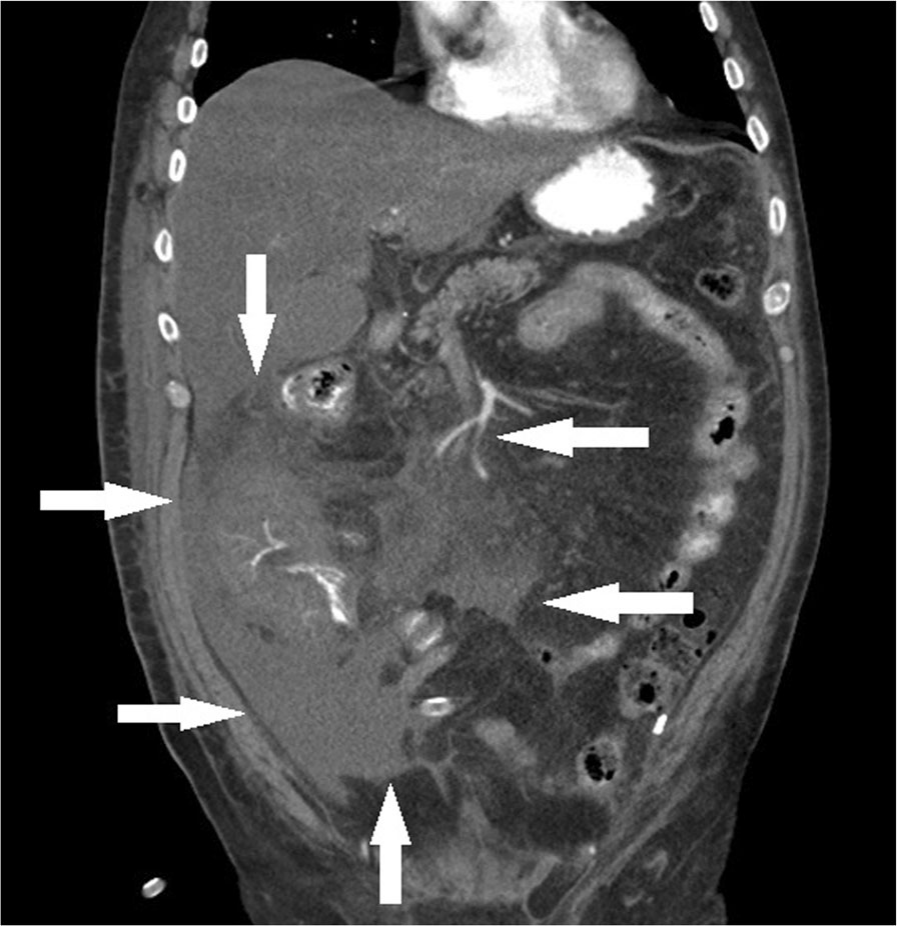
CT scan showing an infiltrate in mesocolon ascendens invading radix mesenterii and the mesenteric vessels

A 65-years old previously fit and well Caucasian male patient presented to a small district general hospital with a 3 weeks history of recurrent rigors, fevers and night sweats. He was found to have kidney injury and thrombocytopenia. The whole-body CT (computed tomography) scan revealed in the retroperitoneal space a lesion measuring approx. 13x8x8cm (Fig. 1). The patient was referred to our hospital for further evaluation and treatment. At presentation, blood biochemistry showed low platelets (55 × 10^9^/L, reference range (ref.) 150–400), low white blood cells (3.2 × 10^9^/L, ref. 4.0–10.0) with lymphocytopenia (0.47 × 10^9^/l, ref. 0.8–4.0). The liver enzymes were elevated, too (Alanine Aminotransferase 1.16μkat/l, ref. < 0.73; Aspartate Aminotransferase 3.56μkat/l, ref. < 0.67) and albumin was low (21.9 g/l, ref. 35.0–53.0). The patient underwent explorative laparotomy, which revealed a tumor in mesocolon ascendens invading radix mesenterii. A surgical biopsy was performed. Few days later, he developed progressive pancytopenia, coagulopathy (fibrinogen 0,7 g/l) and his serum C-reactive protein concentration increased (140 mg/l). The patient was readmitted to the intensive care unit and treated with platelet transfusions, fibrinogen, prothrombin complex concentrates and broad-spectrum antibiotics. Despite all these measures, he developed multi-organ failure with dominant liver failure (Aspartate aminotransferase 18.64 μkat/l, ref. < 0.67; Bilirubin 158.4 μmol/l, ref. < 21.0) and refractory shock. He died 22 days after the initial presentation. At the time of death, the underlying disease leading to death was not known. An autopsy was performed.

### Biopsy findings

**Fig. 2:**
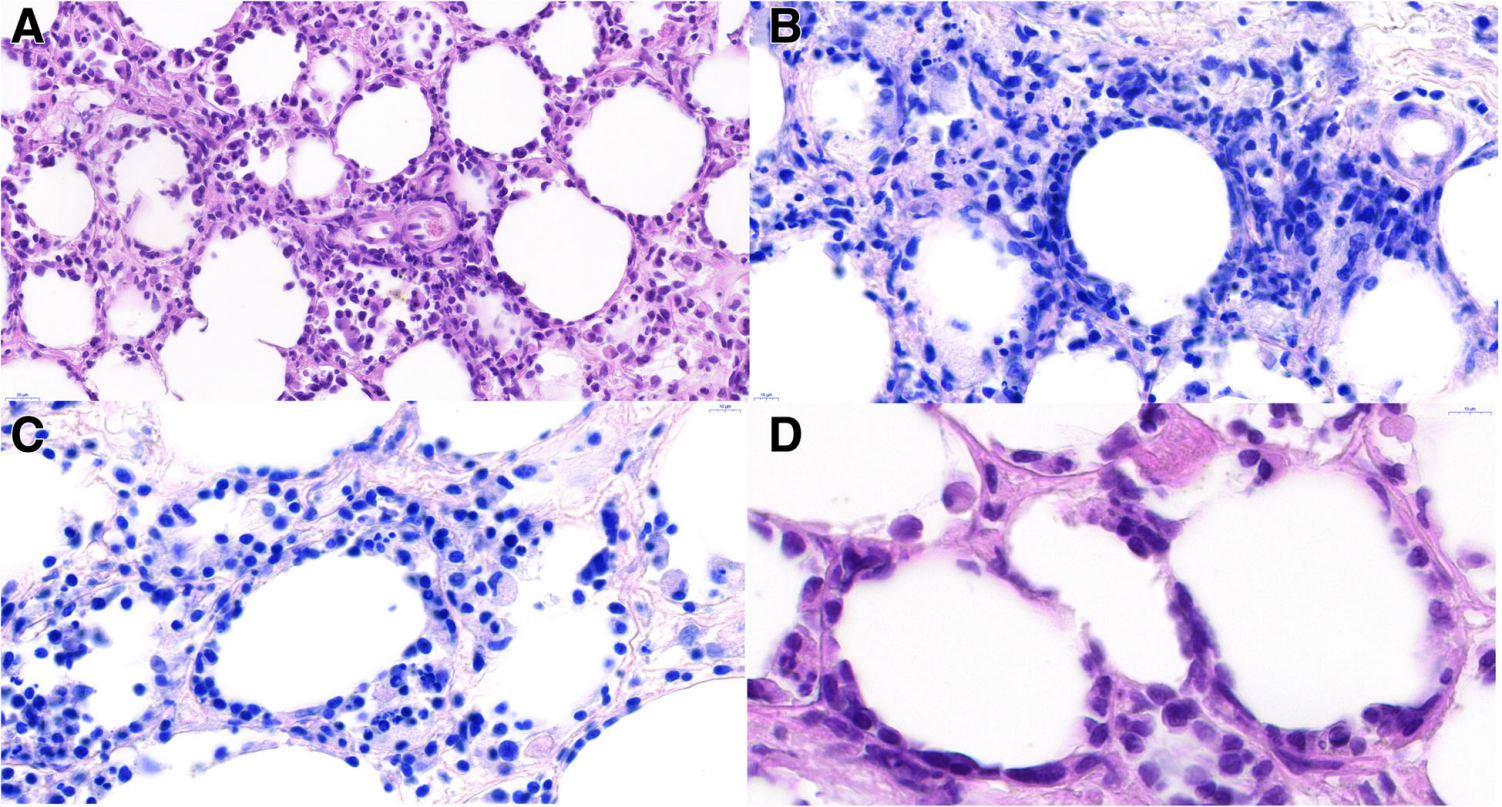
scans of histological slides from the mesenterial fat tissue showing dense lymphocytic infiltration consisting mostly of medium-sized cells with hyperchromatic irregular nuclei. Note a pattern of isolated adipocytes surrounded by a rim of lymphocytes (“rimming”) and large macrophages engulfing lymphocytes (hemophagocytosis). A – HE 60x, B – Giemsa 85x, C – Giemsa 105x, D – HE 145x

**Fig. 3:**
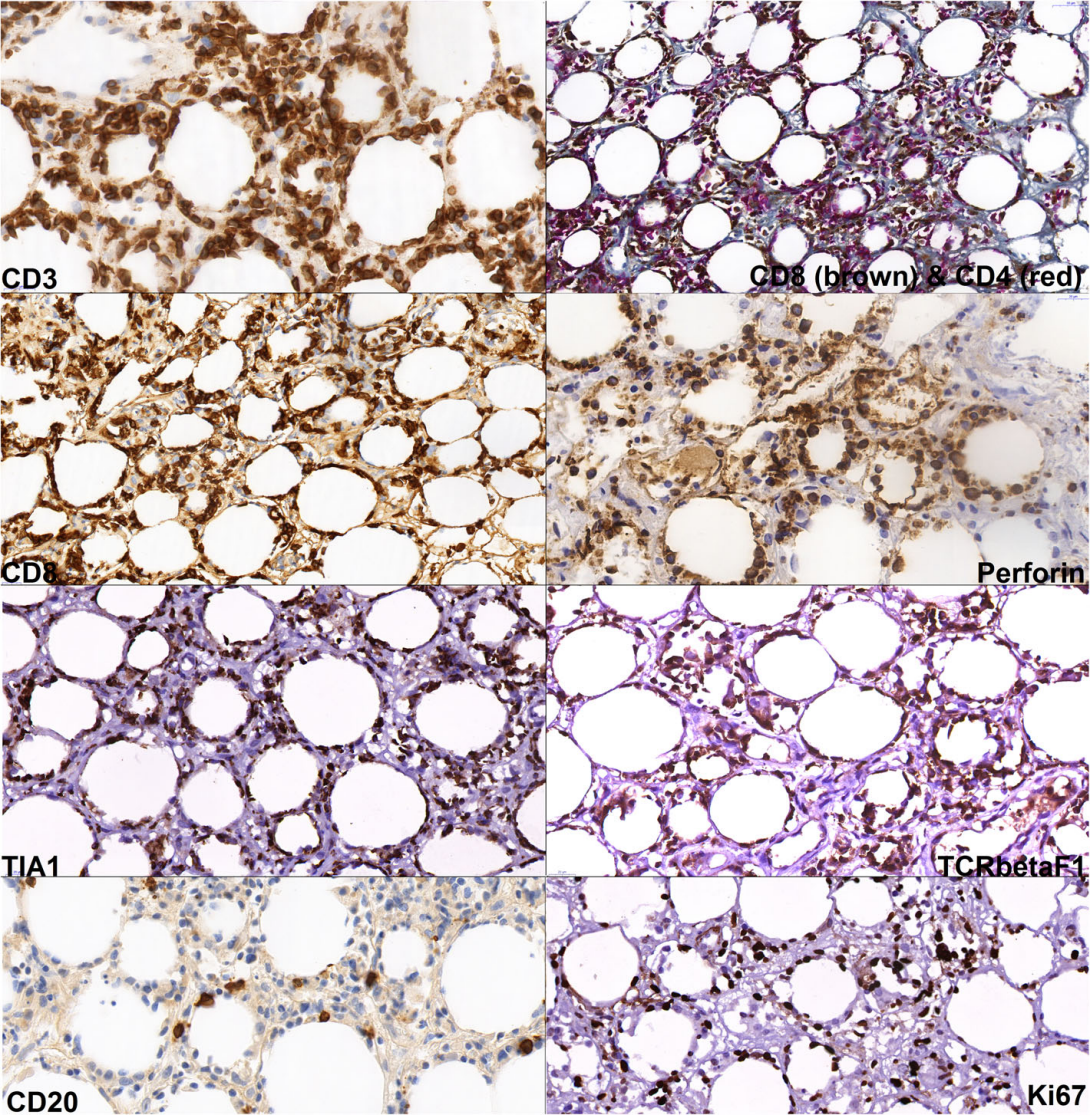
immunohistochemistry showing neoplastic lymphocytes with positivity of CD3, CD8, perforin, TIA-1 and TCRβF1. CD4 stains rather bystander T-cells and histiocytes, without unequivocal positivity in lymphoma. The neoplastic cells are CD20 negative, note the sparse CD20-positive reactive B-cells. The neoplastic lymphocytes embody proliferation activity (Ki67) about 50%

Two formalin-fixed adipose tissue fragments with the size of 16x8x4 mm and 12x7x3 mm were sent to the Department of Pathology. Microscopically, we saw adult fat tissue with dense lymphocytic infiltration. The lymphocytic infiltration consisted mostly of small- to medium-sized cells with hyperchromatic irregular nuclei with little rim of pale cytoplasm. There was a pattern of isolated adipocytes surrounded by a dense rim of hyperchromatic lymphocytes (Fig. 2). We also found necrotic adipocytes and reactive macrophages phagocytizing lymphocytes and erythrocytes. Immunohistochemically, the atypical cells rimming the adipocytes stained for CD45(LCA), CD2, CD3, CD5, CD7, CD8, granzyme B, perforin, TIA1 and TCRβF1 and did not stain for CD4, CD20, CD79a, CD56, CD30, EBER, CD1a, S100, myeloperoxidase, cytokeratin CAM5.2 and TCRγ. The proliferation index Ki67 varied between 10 and 50% (Fig. 3).

Genomic deoxyribonucleic acid (DNA) from formalin-fixed paraffin-embedded (FFPE) tissue section was isolated using the QIAamp DNA FFPE Tissue Kit (Qiagen GmbH, Hilden, Germany). The clonality of the TCR rearrangements (TCR β, γ and δ) were tested using standardized multiplex polymerase chain reaction (PCR), as described by the BIOMED2 study group [8]. The clonality of the PCR products was assessed using an Agilent 2100 Bioanalyzer (Agilent Technologies, Santa Clara, CA, USA), with detection of clonal rearrangement of TCR-γ. Tests for clonal rearrangement in TCR-β and TCR-δ were negative.

Based on these morphological, immunohistochemical and molecular findings, we diagnosed panniculitic T-cell lymphoma with morphological and immunohistochemical features of SPTCL.

### Autopsy finding

During outer inspection we found yellowish color of entire skin and cutaneous suffusions in the abdominal and genital regions. During the inner inspection, there was a thickened, firm, whitish area approx. 12x10x10 cm in the mesocolon ascendens. Intestine and mesenteric lymph nodes were unremarkable. Spleen was enlarged (470 g) and of soft consistency without focal lesions. Liver (2100 g) had a blunt margin, soft consistency and yellow cut surface. Other organs were unremarkable on gross examination. We collected samples for histopathological examination. We specifically and thoroughly examined subcutaneous adipose tissue and looked for any signs of a tumor. We have only found superficial reddish spots that were also sampled for microscopy.

**Fig. 4:**
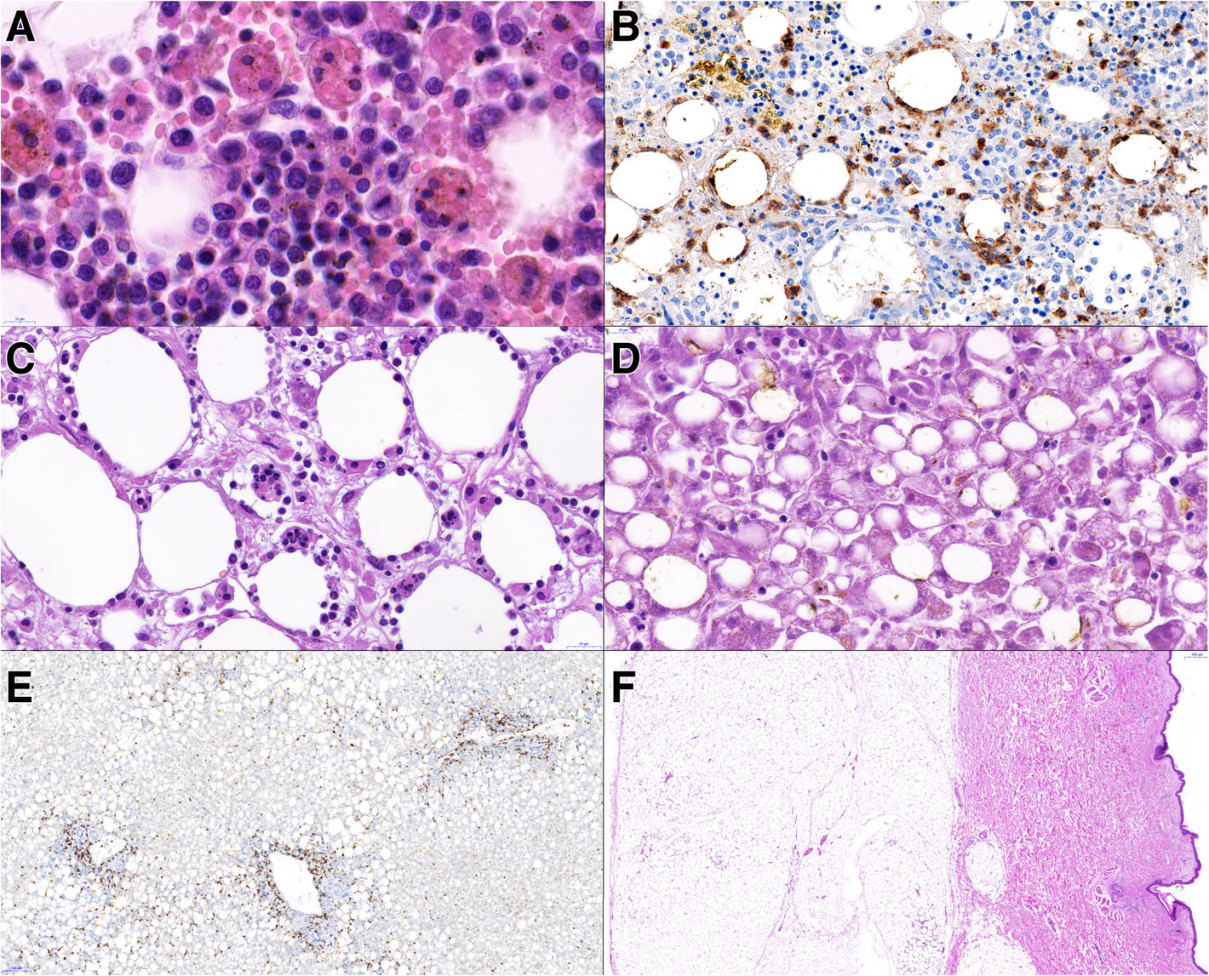
scans of histological slides from the autopsy. A – vertebral bone marrow with large macrophages engulfing red cells and lymphocytes (hemophagocytosis). HE, 130x. B – vertebral bone marrow with focal finding of CD8+ lymphocytes rimming the adipocytes; the finding is highly suspect from infiltration by lymphoma, CD8 immunohistochemistry, 55x. C – mesentery with macrophages engulfing lymphocytes, HE, 73x. D – liver tissue showing marked dystrophic changes including steatosis and cholestasis of hepatocytes. HE, 70x. E – liver tissue without apparent lymphoma infiltration. CD3 immunohistochemistry, 12x. F – sample from skin and subcutis tissue without apparent lymphoma infiltration. HE, 2,5x

Histopathological examination of mesenterial fat tissue revealed similar findings to those described above in the biopsy: adult fat tissue with atypical hyperchromatic lymphoid cells rimming the adipocytes, with macrophages engulfing whole lymphocytes and erythrocytes (Fig. 4c). In the spleen there was marked activation of red pulp with abundant histiocytes but no signs of infiltration by lymphoma. The bone marrow had cellularity about 50%, with trilinear hematopoiesis, with admixture of macrophages with signs of hemophagocytosis of lymphocytes and red blood cells (Fig. 4a). The immunohistochemistry of the vertebral bone marrow showed focal infiltration of CD8+ lymphocytes rimming the adipocytes; the finding is highly suspect from presence of lymphoma cells in the bone marrow (Fig. 4b). Liver tissue showed marked steatosis with cholestatic features (Fig. 4d), but no signs of lymphoma (Fig. 4e). Histopathological examination of the other organs, including abdominal skin and subcutaneous fat tissue (Fig. 4f), was unremarkable.

The autopsy determined the underlying disease as T-cell lymphoma with cytotoxic phenotype, with morphological and immunohistochemical features of SPTCL, associated with signs of hemophagocytic syndrome (clinical findings of fever, bicytopenia and hypofibrinogenemia, autoptic finding of enlarged liver and spleen and histiocytic hemophagocytosis of lymphocytes and red blood cells), probably leading to patients death due to multiorgan failure, in line with reported clinical features.

## Discussion

Subcutaneous panniculitis-like T cell lymphoma (SPTCL) was first described by Gonzalez et al. in 1991 [9]. In the largest reported cohort study of SPTCL performed by Willemze et al., 83 cases had been reviewed. None of these had evidence of lymphoma outside the subcutaneous tissue [3], but there were few case reports about mesenterial involvement and simultaneous subcutaneous involvement [5, 6]. To the best of our knowledge, a case of panniculitic T-cell lymphoma with morphological and immunohistochemical features of SPTCL constituting mesenterial mass without involvement of subcutaneous fat, was not yet reported in medical literature.

The histopathological differential diagnosis of SPTCL includes lupus erythematosus panniculitis (LEP, lupus profundus), however, reports of the mesenterial localization of this are exceedingly rare [10]. Clinically, LEP and SPTCL are indistinguishable [11]. According Massone et al., the histopathological features of LEP include presence of B-cell follicles and of plasma cells within the inflammatory infiltrate, septal fat tissue involvement with fibrosis and occasionally presence of eosinophils. In contrast, the most useful features for the diagnosis of SPTCL are the presence of hyperchromatic CD8+ T-lymphocytes and the absence of septal fibrosis, B-cell follicles and plasma cells. Cytotoxic CD8+ T-lymphocytes may be observed in cases of LEP but never constitute the majority of the infiltrate as in SPTCL [11]. In the series of the same authors, all cases of LEP revealed a polyclonal pattern of the TCR-γ gene rearrangement [11]. The PCR analysis may be a feature helpful in the differentiation of LEP from SPTCL. In our case, absence of other lupus signs (i.e. cutaneous involvement), histopathological and molecular findings described above lead us to be confident in our diagnosis of T-cell lymphoma.

Contemporary differential diagnosis of lymphomas is vitally dependent on molecular features. In terms of the present World Health Organization (WHO)-defined categories, our case would best fit into SPTCL because of histology and the presence of major alpha-beta T-cell phenotype (TCRβF1+, CD8+, granzyme B+, perforin +, TIA1+, CD56-, TCR-γ-). However, SPTCL should primarily affect the skin, should have a benign clinical course and unlike gamma-delta phenotypes, only < 20% is associated with hemophagocytic syndrome [3, 12]. Indeed, it can never be ruled out that a subcutaneous lesion was present and missed during autopsy. We consider this very unlikely, because the pathologist performing the autopsy was aware of the biopsy finding and subcutaneous lesions were actively sought for and any suspicious nodules were excised and thoroughly microscopically examined.

According the WHO classification, the term SPTCL is reserved for α/β T-cell phenotype (but not TCRα/β-rearrangement) lymphomas containing CD8+, granzyme B+, perforin+, TIA1+, CD4- and CD56- cells, which is limited to subcutaneous tissue (no dermal and/or epidermal involvement) and bears relatively good prognosis due to a good response to conservative immunosuppressive regimens. SPTCL is distinct from primary cutaneous γ/δ T-lymphomas, which are typically CD4-, CD8-, CD56+, granzyme B+, perforin+, TIA1+, may involve the epidermis and/or dermis [13, 14], may present with panniculitic pattern [15] and invariably have a very poor prognosis [1]. Studies [1, 3, 4, 14–21] showed that TCR α/β lymphomas represent often an indolent disease, whilst γ/δ phenotype harbors a poor prognosis. In the study by Toro et al., median survival was 15 vs. 166 months; 5-years survival 10% vs. 80% in α/β vs. γ/δ T-cell phenotype, respectively [21]. Similar 5-years survival rates were reported by Willemze et al.: 11% vs. 82% [3].

The γ/δ-TCR-rearranged lymphomas are more frequently associated with hemophagocytic syndrome [3], this association was first described by Avionach et al. in 1994 [22]. The hemophagocytic syndrome (hemophagocytic lymphohistiocytosis, HLH) represents a severe hyperinflammatory disease with prolonged fever, cytopenias, hepatosplenomegaly, and hemophagocytosis by activated non-neoplastic macrophages [23]. The diagnosis of HLH is based on presence of at least five of these eight signs: fever, splenomegaly, bicytopenia, hypertriglyceridemia and/or hypofibrinogenemia, hemophagocytosis, low/absent natural-killer-cell-activity, hyperferritinemia, and high-soluble interleukin-2-receptor levels [24]. Our patient had fever, hepatosplenomegaly, bicytopenia, hypofibrinogenemia and histopathological finding of hemophagocytosis. Plasma triglycerides, ferritin and high-soluble interleukin-2-receptor were not measured.

Frequent non-neoplastic triggers of HLH are infectious agents, mostly viruses of the herpes group, or rheumatic diseases [23]. There are multiple reports of HLH associated with subcutaneous T-cell lymphomas [8, 9, 25–27]. Concerning tumors triggering HLH, the most common are hematological neoplasms (93%), more frequently T-cell than B-cell lymphomas or leukemias, and only rarely solid tumors [28–30]. The pathogenesis of HLH is related to deranged immune response. Dysfunctional cytotoxic CD8+ T lymphocytes (CTLs) and NK cells are unable to initiate appropriate response against malignant or infected cells. Histiocytes proliferate, produce storm of cytokines, invade liver, spleen and lymph nodes, and engulf blood cells and platelets [31]. The immune system is unable to control the hyperinflammatory response, which often leads to multiorgan failure and death.

## Conclusion

In conclusion, we described a patient who died of a hemophagocytic syndrome accompanying a mesenteric tumor with morphologic and molecular features of SPTCL without clinical or morphological involvement of subcutaneous tissue.

## Data Availability

It is not possible to share research data publicly.

## Abbreviations

CD: Cluster of differentiation
CT: Computer tomography
CTLs: Cytotoxic T-lymphocytes
DNA: Deoxyribonucleic acid
FFPE: Formalin-fixed paraffin-embedded
HLH: Hemophagocytic lymphohistiocytosis
INF-γ: Interferon-γ
LEP: Lupus erythematosus panniculitis
PCR: Polymerase chain reaction
Ref.: Reference range
SPTCL: Sub cutaneous panniculitis-like T-cell lymphoma
TCR: T-cell receptor
TIA1: T-cell intracellular antigen 1
